# Supplementary material for: Bio-Rad and QIAGEN digital PCR platforms provide equivalent quantification for wastewater-based SARS-CoV-2 surveillance
Source: Appl Environ Microbiol. 2026 Apr 22;92(5):e00083-26. doi: 10.1128/aem.00083-26 (PMC13188893; doi:10.1128/aem.00083-26)
Supplement: Supplemental material — Figures S1 to S6; Tables S1 to S13. [file aem.00083-26-s0001.docx]

# Supplemental Materials

This supplemental material supports the manuscript: “Equivalent Performance of Bio-Rad and QIAGEN Digital PCR Platforms for SARS-CoV-2 Wastewater Surveillance”.

## Supplemental Figures

#### Figure S1. Optimized KingFisher Flex extraction protocol for wastewater samples on MCE filters using BioMérieux EasyMag reagents.

(see next page)

| Tip1 | |  | 96 DW tip comb | | | | |
| --- | --- | --- | --- | --- | --- | --- | --- |
| Pick-Up | | Tip Comb | |  | | |  |
| Binding 1 | | Lysate 1 | |  | | |  |
| Beginning of step | | Precollect | | No | | |  |
|  | | Release time, speed | | 00:00:20, Medium | | |  |
| Mixing / heating: | | Mixing time, speed | | 00:03:20, Medium | | |  |
|  | | Heating during mixing | | No | | |  |
| End of step | | Postmix | | No | | |  |
|  | | Collect count | | 5 | | |  |
|  | | Collect time [s] | | 25 | | |  |
| Collect Beads-1 | | Lysate 1 | |  | | |  |
| Beginning of step | | Precollect | | No | | |  |
|  | | Release beads | | No | | |  |
| Mixing / heating: | | Mixing time, speed | | 00:02:30, Slow | | |  |
|  | | Heating during mixing | | No | | |  |
| End of step | | Postmix | | No | | |  |
|  | | Collect beads | | No | | |  |
| Wash 1 | | Wash 1 | |  | | |  |
| Beginning of step | | Precollect | | No | | |  |
|  | | Release time, speed | | 00:00:20, Medium | | |  |
| Mixing / heating: | | Mixing time, speed | | 00:00:30, Medium | | |  |
|  | | Heating during mixing | | No | | |  |
| End of step | | Postmix | | No | | |  |
|  | | Collect count | | 3 | | |  |
|  | | Collect time [s] | | 2 | | |  |
| Wash 2 | | Wash 2 | |  | | |  |
| Beginning of step | | Precollect | | No | | |  |
|  | | Release beads | | Yes | | |  |
| Mixing / heating: | | Mixing time, speed | | 00:00:30, Medium | | |  |
|  | | Heating during mixing | | No | | |  |
| End of step | | Postmix | | No | | |  |
|  | | Collect count | | 3 | | |  |
|  | | Collect time [s] | | 2 | | |  |
| Wash 3 | | Wash 3 | |  | | |  |
| Beginning of step | Precollect | | No | |  |  |  |
|  | Release beads | | Yes | |  |  |  |
| Mixing / heating: | Mixing time, speed | | 00:00:30, Medium | |  |  |  |
|  | Heating during mixing | | No | |  |  |  |
| End of step | Postmix | | No | |  |  |  |
|  | Collect count | | 3 | |  |  |  |
|  | Collect time [s] | | 2 | |  |  |  |
| Wash 4 | | | Wash 4 | |  | |  |
| Beginning of step | | | Precollect | | No | |  |
|  | | | Release beads | | Yes | |  |
| Mixing / heating: | | | Mixing time, speed | | 00:00:15, Slow | |  |
|  | | | Heating during mixing | | No | |  |
| End of step | | | Postmix | | No | |  |
|  | | | Collect count | | 4 | |  |
|  | | | Collect time [s] | | 3 | |  |
| Wash 5 | | | Wash 5 | |  | |  |
| Beginning of step | | | Precollect | | No | |  |
|  | | | Release beads | | Yes | |  |
| Mixing / heating: | | | Mixing time, speed | | 00:00:15, Slow | |  |
|  | | | Heating during mixing | | No | |  |
| End of step | | | Postmix | | No | |  |
|  | | | Collect count | | 4 | |  |
|  | | | Collect time [s] | | 3 | |  |
| Elution | | | Elution | |  | |  |
| Beginning of step | | | Precollect | | No | |  |
|  | | | Release beads | | Yes | |  |
| Mixing / heating: | | | Mixing time, speed | | 00:05:00, Medium | |  |
|  | | | Heating temperature [°C] | | 60 | |  |
|  | | | Preheat | | Yes | |  |
| End of step | | | Postmix | | No | |  |
|  | | | Collect count | | 4 | |  |
|  | | | Collect time [s] | | 3 | |  |
| Collect Beads | | | Elution | |  | |  |
| Beginning of step | | | Precollect | | No | |  |
|  | | | Release beads | | No | |  |
| Mixing / heating: | | | Mixing time, speed | | 00:01:30, Slow | |  |
|  | | | Heating during mixing | | No | |  |
| End of step | | | Postmix | | No | |  |
|  | | | Collect beads | | No | |  |
| Leave | | | Tip Comb | |  | |  |

#### Figure S2. Plate layouts for extraction, reverse transcription, and digital PCR analyses.

|  | **EXTRACT/RT Plate 1** | | | | | | | | | | | |
| --- | --- | --- | --- | --- | --- | --- | --- | --- | --- | --- | --- | --- |
|  | 1 | 2 | 3 | 4 | 5 | 6 | 7 | 8 | 9 | 10 | 11 | 12 |
| A | 1 | 9 | 17 | 25 | 33 | 41 | NEC | - | - | - | - | - |
| B | 2 | 10 | 18 | 26 | 34 | 42 | NEC | - | - | - | - | - |
| C | 3 | 11 | 19 | 27 | 35 | 43 | POS | - | - | - | - | - |
| D | 4 | 12 | 20 | 28 | 36 | 44 | POS | - | - | - | - | - |
| E | 5 | 13 | 21 | 29 | 37 | 45 | NTC | - | - | - | - | - |
| F | 6 | 14 | 22 | 30 | 38 | 46 | NTC | - | - | - | - | - |
| G | 7 | 15 | 23 | 31 | 39 | 47 | NTC | - | - | - | - | - |
| H | 8 | 16 | 24 | 32 | 40 | 48 | NTC | - | - | - | - | - |
|  |  |  |  |  |  |  |  |  |  |  |  |  |
|  |  |  |  |  |  |  |  |  |  |  |  |  |
|  | **EXTRACT/RT Plate 2** | | | | | | | | | | | |
|  | 1 | 2 | 3 | 4 | 5 | 6 | 7 | 8 | 9 | 10 | 11 | 12 |
| A | 49 | 57 | 65 | 73 | 81 | 89 | nec | - | - | - | - | - |
| B | 50 | 58 | 66 | 74 | 82 | 90 | nec | - | - | - | - | - |
| C | 51 | 59 | 67 | 75 | 83 | 91 | pos | - | - | - | - | - |
| D | 52 | 60 | 68 | 76 | 84 | 92 | pos | - | - | - | - | - |
| E | 53 | 61 | 69 | 77 | 85 | 93 | ntc | - | - | - | - | - |
| F | 54 | 62 | 70 | 78 | 86 | 94 | ntc | - | - | - | - | - |
| G | 55 | 63 | 71 | 79 | 87 | 95 | ntc | - | - | - | - | - |
| H | 56 | 64 | 72 | 80 | 88 | 96 | ntc | - | - | - | - | - |
|  |  |  |  |  |  |  |  |  |  |  |  |  |

|  | **N1 and *hepG* Plate 1** | | | | | | | | | | | |
| --- | --- | --- | --- | --- | --- | --- | --- | --- | --- | --- | --- | --- |
|  | 1 | 2 | 3 | 4 | 5 | 6 | 7 | 8 | 9 | 10 | 11 | 12 |
| A | 1 | 1 | 1 | 9 | 9 | 9 | 17 | 17 | 17 | NEC | - | - |
| B | 2 | 2 | 2 | 10 | 10 | 10 | 18 | 18 | 18 | NEC | - | - |
| C | 3 | 3 | 3 | 11 | 11 | 11 | 19 | 19 | 19 | POS | - | - |
| D | 4 | 4 | 4 | 12 | 12 | 12 | 20 | 20 | 20 | POS | - | - |
| E | 5 | 5 | 5 | 13 | 13 | 13 | 21 | 21 | 21 | NTC | - | - |
| F | 6 | 6 | 6 | 14 | 14 | 14 | 22 | 22 | 22 | NTC | - | - |
| G | 7 | 7 | 7 | 15 | 15 | 15 | 23 | 23 | 23 | NTC | - | - |
| H | 8 | 8 | 8 | 16 | 16 | 16 | 24 | 24 | 24 | NTC | - | - |
|  |  |  |  |  |  |  |  |  |  |  |  |  |
|  | **N1 and *hepG* Plate 2** | | | | | | | | | | | |
|  | 1 | 2 | 3 | 4 | 5 | 6 | 7 | 8 | 9 | 10 | 11 | 12 |
| A | 25 | 25 | 25 | 33 | 33 | 33 | 41 | 41 | 41 | NEC | - | - |
| B | 26 | 26 | 26 | 34 | 34 | 34 | 42 | 42 | 42 | NEC | - | - |
| C | 27 | 27 | 27 | 35 | 35 | 35 | 43 | 43 | 43 | POS | - | - |
| D | 28 | 28 | 28 | 36 | 36 | 36 | 44 | 44 | 44 | POS | - | - |
| E | 29 | 29 | 29 | 37 | 37 | 37 | 45 | 45 | 45 | NTC | - | - |
| F | 30 | 30 | 30 | 38 | 38 | 38 | 46 | 46 | 46 | NTC | - | - |
| G | 31 | 31 | 31 | 39 | 39 | 39 | 47 | 47 | 47 | NTC | - | - |
| H | 32 | 32 | 32 | 40 | 40 | 40 | 48 | 48 | 48 | NTC | - | - |
|  |  |  |  |  |  |  |  |  |  |  |  |  |

|  | **N1 and *hepG* Plate 3** | | | | | | | | | | | |
| --- | --- | --- | --- | --- | --- | --- | --- | --- | --- | --- | --- | --- |
|  | 1 | 2 | 3 | 4 | 5 | 6 | 7 | 8 | 9 | 10 | 11 | 12 |
| A | 49 | 49 | 49 | 57 | 57 | 57 | 65 | 65 | 65 | NEC | - | - |
| B | 50 | 50 | 50 | 58 | 58 | 58 | 66 | 66 | 66 | NEC | - | - |
| C | 51 | 51 | 51 | 59 | 59 | 59 | 67 | 67 | 67 | POS | - | - |
| D | 52 | 52 | 52 | 60 | 60 | 60 | 68 | 68 | 68 | POS | - | - |
| E | 53 | 53 | 53 | 61 | 61 | 61 | 69 | 69 | 69 | NTC | - | - |
| F | 54 | 54 | 54 | 62 | 62 | 62 | 70 | 70 | 70 | NTC | - | - |
| G | 55 | 55 | 55 | 63 | 63 | 63 | 71 | 71 | 71 | NTC | - | - |
| H | 56 | 56 | 56 | 64 | 64 | 64 | 72 | 72 | 72 | NTC | - | - |
|  |  |  |  |  |  |  |  |  |  |  |  |  |
|  | **N1 and *hepG* Plate 4** | | | | | | | | | | | |
|  | 1 | 2 | 3 | 4 | 5 | 6 | 7 | 8 | 9 | 10 | 11 | 12 |
| A | 73 | 73 | 73 | 81 | 81 | 81 | 89 | 89 | 89 | NEC | - | - |
| B | 74 | 74 | 74 | 82 | 82 | 82 | 90 | 90 | 90 | NEC | - | - |
| C | 75 | 75 | 75 | 83 | 83 | 83 | 91 | 91 | 91 | POS | - | - |
| D | 76 | 76 | 76 | 84 | 84 | 84 | 92 | 92 | 92 | POS | - | - |
| E | 77 | 77 | 77 | 85 | 85 | 85 | 93 | 93 | 93 | NTC | - | - |
| F | 78 | 78 | 78 | 86 | 86 | 86 | 94 | 94 | 94 | NTC | - | - |
| G | 79 | 79 | 79 | 87 | 87 | 87 | 95 | 95 | 95 | NTC | - | - |
| H | 80 | 80 | 80 | 88 | 88 | 88 | 96 | 96 | 96 | NTC | - | -ß |

#### Figure S3. Representative fluorescence amplitude plots demonstrating threshold positioning.

Screenshots from the QX200 and the QIAcuity readout demonstrating threshold positioning. (A) is an image of the partitioning observed for the N1 target using QuantaSoft (Bio-Rad). Notice multiple populations of N1 due to mutations in the viral genome since the original primer design in 2019. (B) demonstrates an image visualized using QuantaSoft for PCR inhibition control, *gyrA* gene.

**(A) N1**


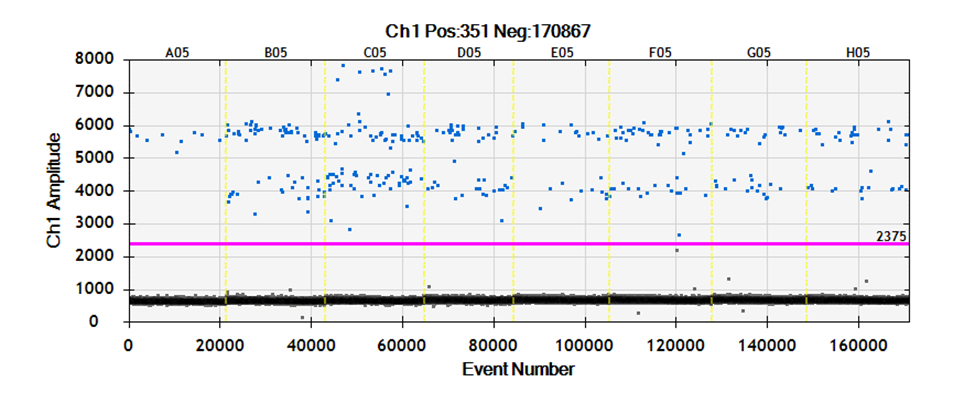


**(B) *gyrA* gene**


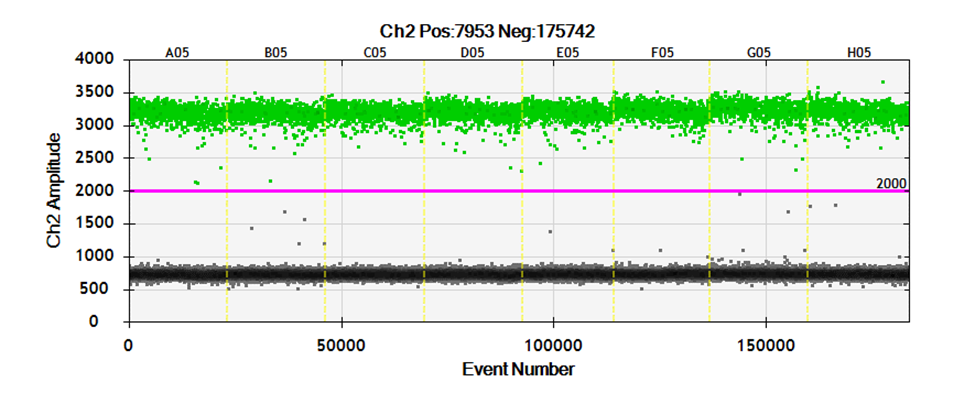


#### Figure S4. Single-well (non-hyperwelled) concentration comparison between platforms across concentration bins.


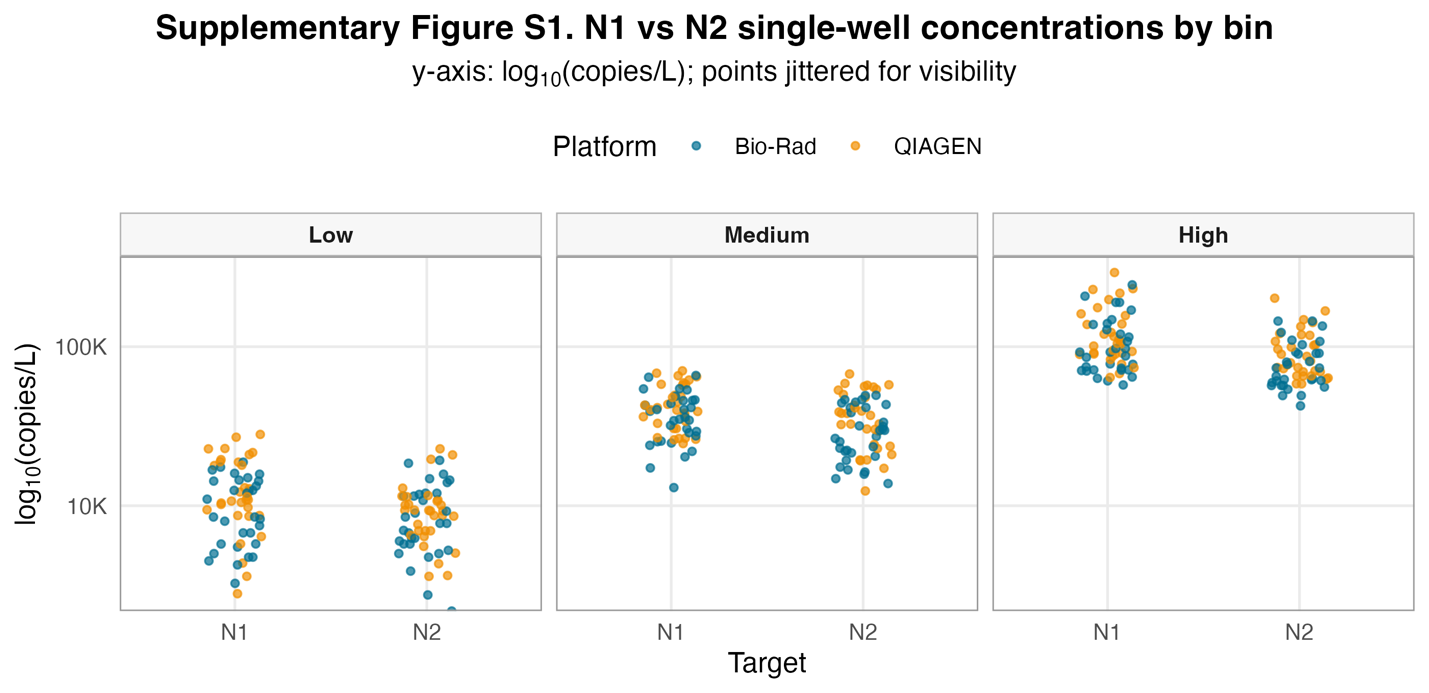


#### Figure S5. Single-well (non-hyperwelled) concentration comparison between concentration bins across platforms.


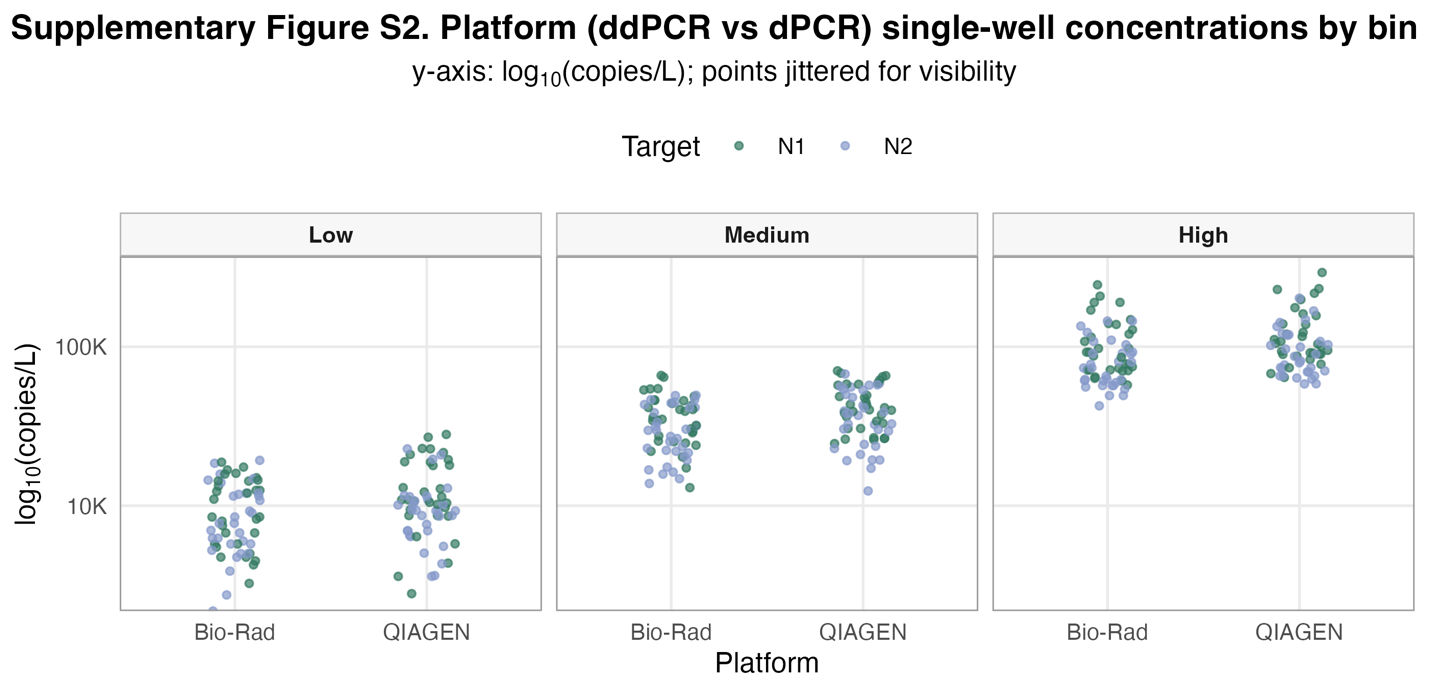


#### Figure S6. Model 1 residual diagnostics assessing dataset normality.


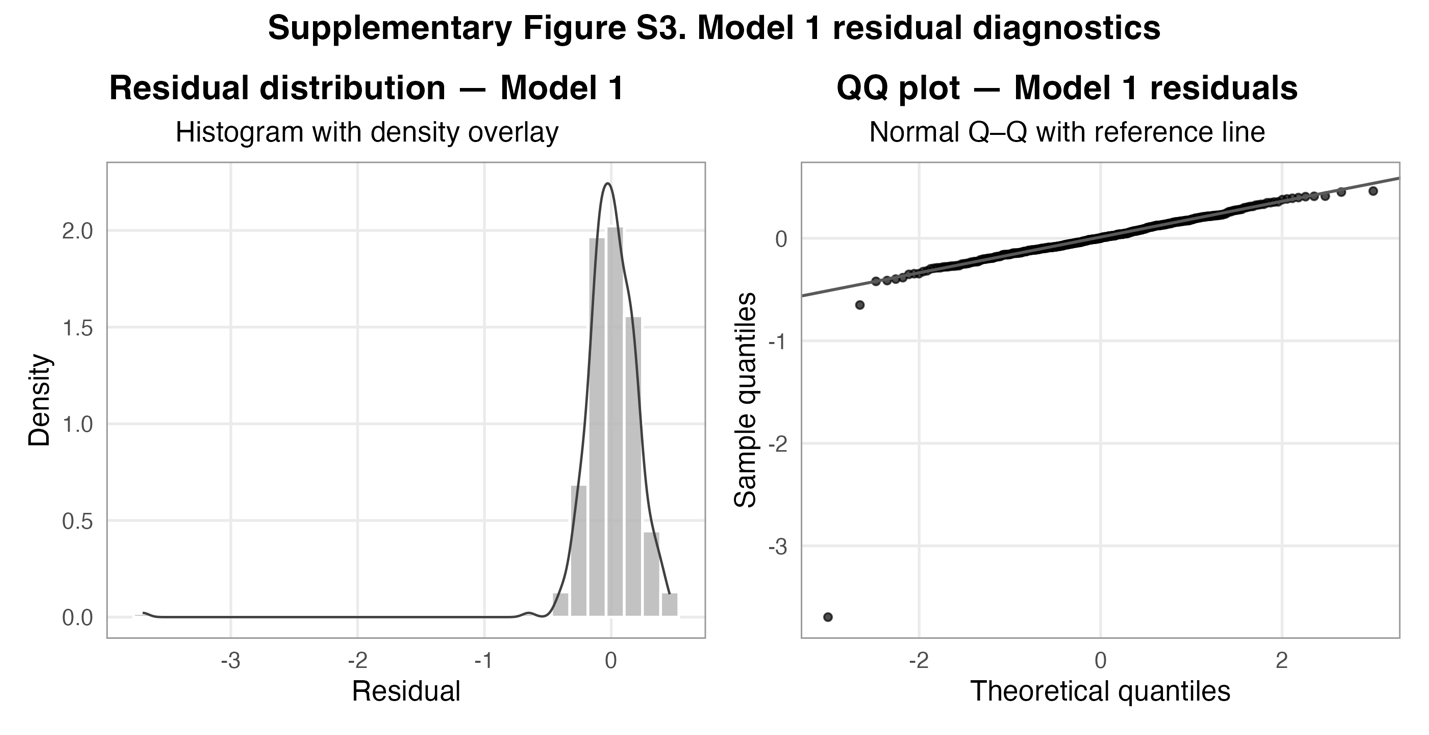


## Supplemental Tables

### GROUP 1: Extraction and Sample Processing

#### Table S1. Reagent volumes and catalog information for total nucleic acid extraction on KingFisher^TM^ Flex.

| Quantity | Description | Vendor | Catalog Number |
| --- | --- | --- | --- |
| 1 | Tip comb | Fisher Sci. | 22-387-029 |
| 1 | 96 well plate | Bio-Rad | 12001925 |
| 8 | 96 well, DWP | Fisher Sci. | 22-387-031 |
| 1mL | Lysis Buffer | BioMérieux |  |
| 800µL | Wash Buffer 1 | BioMérieux | 280130 |
| 1000µL | Wash Buffer 2 | BioMérieux | 280131 |
| 500µL | Wash Buffer 3 | BioMérieux | 280132 |
| 50µL | Magnetic silica | BioMérieux | 280134 |
| 100µL | Buffer AE | Life Technologies | 19077 |

Table S2. KingFisher^TM^ Flex plate preparation and setup instructions.

The following describes the setup of the necessary plates to be prepared prior to the initiation of the extraction on the KingFisher Flex. Plates should be labeled with the appropriate plate name on the short side opposite well A1 of a 96 DWP. The appropriate volume of reagents should be added to a reagent reservoir and individual well volumes dispensed with a multichannel pipette.

| Plate name | Reagent | Total volume needed for 96 extractions | Volume per well | Instructions |
| --- | --- | --- | --- | --- |
| Lysate* | Magnetic silica beads (well mixed) | 5mL | 50µL | Incubate lysate with magnetic beads for 10 minutes at room temperature |
| Wash 1 | Wash Buffer 1 | 40mL | 400µL |  |
| Wash 2 | Wash Buffer 1 | 40mL | 400µL |  |
| Wash 3 | Wash Buffer 2 | 50mL | 500µL |  |
| Wash 4 | Wash Buffer 2 | 50mL | 500µL |  |
| Wash 5 | Wash Buffer 3 | 50mL | 500µL |  |
| Eluate | Buffer AE | 10mL | 100µL |  |
| Tip Comb |  |  |  | Place 96 DW tip comb into plate |

**Containing 50µL of magnetic silica beads and up to 950µL of lysed sample supernatant.*

### GROUP 2: Primer, Probes, and Assay Conditions

#### Table S3. Primer and probe sequences for SARS-CoV-2 targets and quality control elements.

Primer and probe sequences used in this study. Primers and probes were purchased from LGC Biosearch unless otherwise noted.

| **Target Gene** | ***Primers and Probes*** | ***Sequences 5'-3'*** | ***Nucleotide Position*** | ***Amplicon length*** | ***R*eference** |
| --- | --- | --- | --- | --- | --- |
| N1 Nucleocapsid | nCoV N1 FWD | GACCCCAAAATCAGCGAAAT^1^ | 28,303-28,322 | 73 bp | 2019-Novel coronavirus (2019-nCoV) real-time rRT-PCR panel primers and probes (30) |
|  | nCoV N1 REV | TCTGGTTACTGCCAGTTGAATCTG^1^ | 28,374-28,351 |  |  |
|  | nCoV N1 FAM Probe | FAM-ACCCCGCATTACGTTTGGTGGACC-BHQ-1^1^ | 28,325-28,348 |  |  |
| N2 Nucleocapsid | nCoV N2 FWD | TTACAAACATTGGCCGCAAA^1^ | 29,180-29,199 | 66 bp | 2019-Novel coronavirus (2019-nCoV) real-time rRT-PCR panel primers and probes (30) |
|  | nCoV N2 REV | GCGCGACATTCCGAAGAA^1^ | 29,246-29,228 |  |  |
|  | nCoV N2 FAM Probe | FAM-ACAATTTGCCCCCAGCGCTTCAG-BHQ-1^1^ | 29,204-29,226 |  |  |
| Bovine Coronavirus | BCoV_F | CTGGAAGTTGGTGGAGTT^1^ | 29,026-29,043 | 85 bp |  |
|  | BCoV_R | ATTATCGGCCTAACATACATC^2^ | 29,090-29,110 |  |  |
|  | BCoV_FAM Probe | FAM-CCTTCATATCTATACACATCAAGTTGTT-BHQ-1^2^ | 29,058-29,085 |  | (31) |
| Halophile | Np_gyra_F | ACGATTACCTGCTCTGCTTTAC^3^ | 1,691964-1,691985 | 120 bp |  |
|  | Np_gyra_R | CGTTGAGGTCGAGAACATTGA^3^ | 1,692083-1,692,063 |  |  |
|  | Np_gyra_HEX | HEX-CAAGGGCAGGTCTATCGGCTGAAG-BHQ-1^3^ | 1,691990-1,692,013 |  |  |
| Hepatitis G | HepG F | CGGCCAAAAGGTGGTGGATG^4^ | 100-119 | 185 bp |  |
|  | HepG R | CGACGAGCCTGACGTCGGG^4^ | 285-267 |  |  |
|  | HepG HEX Probe | HEX-AGGTCCCTCTGGCGCTTGTGGCGAG-BHQ-1^4^ | 172-196 |  | (32) |
| Beta Actin | Mouse ACTB, 20X VIC | Proprietary, Life Technologies |  |  |  |

*^1^ Nucleotide numbering based on SARS-CoV-2 accession no. MN980947.*

*^2^ Nucleotide numbering based on BCoV strain Mebus accession no. U00735.*

*^3^ Nucleotide numbering based on Natronomonas pharaonis DSM 2160 accession no. GCA_000026045.*

*^4^ Nucleotide numbering based on HGV accession no. U4402.*

#### Table S4. Reverse transcription thermal cycling conditions using Reliance Select cDNA Synthesis Kit on the C1000 Touch.

| Step | Temperature (˚C) | Time |
| --- | --- | --- |
| Activate Reverse Transcriptase | 50 | 20 minutes |
| Inactivation of Reverse Transcriptase | 95 | 1 minute |
| Hold | 4 | Indefinite |

#### Table S5. Duplexed digital PCR assay design and fluorophore combinations.

| Duplex # | Target | Fluorophore |
| --- | --- | --- |
| 1 | N1 nucleocapsid | FAM |
|  | Hepatitis G | HEX |
| 2 | N2 nucleocapsid | FAM |
|  | Mouse Beta Actin | VIC |
| 3 | Bovine Coronavirus | FAM |
|  | *gyrA* gene | HEX |

#### Table S6. Bio-Rad QX200 thermal cycling conditions for all duplexed assays on the C1000 Touch.

| Step | Temperature (˚C) | Time | Number of cycles | Notes |
| --- | --- | --- | --- | --- |
| Enzyme Activation | 94 | 10 minutes |  |  |
| Denaturation | 94 | 30 seconds | 40 | Ramp rate of 2˚C/second |
| Annealing/Extension | 55 | 60 seconds | 40 |  |
| Enzyme Deactivation | 98 | 10 minutes | 1 |  |
| Hold | 4 | Indefinite | 1 |  |

#### Table S7. QIAGEN QIAcuity thermal cycling conditions.

| Step | Temperature (°C) | Time | Number of Cycles |
| --- | --- | --- | --- |
| Initial Denaturation | 95 | 2 minutes | 1 |
| Denaturation | 95 | 15 seconds | 40 |
| Annealing | 55 | 30 seconds | 40 |
| Imaging | 4 | 10 minutes per nanoplate | -- |

#### Table S8. QIAGEN QIAcuity primer and probe reaction concentrations.

| Assay | Primer Final Reaction Concentration | Probe Final Reaction Concentration |
| --- | --- | --- |
| N1 | 0.4 | 0.4 |
| HepG (duplexed with N1) | 0.4 | 0.2 |
| N2 | 0.8 | 0.8 |
| Mouse Beta Actin (duplexed with N2) | 0.5x | 0.5x |
| BCoV | 0.8 | 0.8 |
| *gyrA* (duplexed with BCoV) | 0.4 | 0.8 |

## GROUP 3: Master Mix Recipes

#### Table S9. Bio-Rax QX200 master mix compositions for duplexed assays

1. **Reverse Transcription Master Mix**

|  | Reliance RT MIX |  |  |  |
| --- | --- | --- | --- | --- |
|  |  | # rxn | 220 |  |
|  |  | per rxn | |  |
|  | **volume per rxn** | 75 | 16500 |  |
|  | **5x Reliance Select cDNA Synthesis Reaction Buffer** | 15 | 3300 |  |
|  | **Reliance Reverse Transcriptase** | 3.75 | 825 |  |
|  | **10x Random Primer Mix** | 7.5 | 1650 |  |
|  | **RNA** | 37.5 | 8250 |  |
|  | **mouse lung RNA** | .5 | 110 |  |
|  | **Water** | 10.75 | 2365 |  |
| 1. **PCR Master Mix for Duplex** | | | | |
| **# of reactions** | **180** |  | **Master Mix** |  |
| **Reagents** | **Final conc** | **Initial vol µL** | **vol µL** |  |
| **Water** |  | 5.35 | 963 |  |
| **2X ddPCR MM** | 1X | 12.5 | 2250 |  |
| **N1 FAM (10 µM)** | 0.25 | 0.625 | 112.5 | **N1 Fam** |
| **N1 FWD (100 µM)** | 0.9 | 0.225 | 40.5 |  |
| **N1 REV (100 µM)** | 0.9 | 0.225 | 40.5 |  |
| **HEPG HEX (10 µM)** | 0.25 | 0.625 | 112.5 | **Hep G HEX** |
| **HEPG FWD (100 µM)** | 0.9 | 0.225 | 40.5 |  |
| **HEG REV (100 µM)** | 0.9 | 0.225 | 40.5 |  |
|  | µl template | 5 | 900 |  |
| **Final Volume** |  | 25 | 4500 |  |

Table S10. QIAGEN QIAcuity master mix compositions for duplexed assays.

| # of Reactions = | 24 | # of Reactions with overage = | 25 |
| --- | --- | --- | --- |
| Reagent | **Final Conc (μM)** | **Initial Vol (µL)** | **Mastermix Vol (µL)** |
| Water |  | 16.2 | 421.2 |
| 4X dPCR Supermix | 1X | 10 | 260 |
| N1 FAM | 0.4 | 1.6 | 41.6 |
| N1 FWD | 0.4 | 1.6 | 41.6 |
| N2 REV | 0.4 | 1.6 | 41.6 |
| HepG HEX | 0.2 | 0.8 | 20.8 |
| HepG FWD | 0.4 | 1.6 | 41.6 |
| HepG REV | 0.4 | 1.6 | 41.6 |
| Template |  | 5 | NA |
| Final Volume |  | 40 | 910 |

### GROUP 4: Statistical Analyses

#### Table S11. Average control results of all samples analyzed in study on both platforms.

| Average Control Results of all Samples Analyzed | | | | |
| --- | --- | --- | --- | --- |
| Platform | **Total Recovery (%)** | **Extraction Recovery (%)** | **PCR Inhibition Efficiency %** | **RT Efficiency (%)** |
| Bio-Rad | 16 | 33 | 95 | 88 |
| QIAcuity | 10 | 24 | 100 | 85 |

#### Table S12. Aligned Rank Transform ANOVA results by concentration bin.

| Bin | Factor | F-value | p-value |
| --- | --- | --- | --- |
| Low | N | 6.00 | 0.0157 |
|  | PCR | 3.42 | 0.0668 |
|  | N:PCR | 1.45 | 0.2312 |
| Medium | N | 7.37 | 0.0076 |
|  | PCR | 7.68 | 0.0065 |
|  | N:PCR | 0.06 | 0.8142 |
| High | N | 16.22 | 0.0001 |
|  | PCR | 7.68 | 0.0065 |
|  | N:PCR | 0.06 | 0.8142 |
| All | N | 4.21 | 0.0409 |
|  | PCR | 2.72 | 0.0999 |
|  | N:PCR | 0.17 | 0.6769 |

### GROUP 5: dMIQE Checklist

#### Table S13. dMIQE checklist

| **ITEM TO CHECK** | **PROVIDED** | **COMMENT** |
| --- | --- | --- |
| **1. SPECIMEN** | | |
| Detailed description of specimen type and numbers | **Y** | Included in main manuscript under "Sample Collection and Processing" |
| Sampling procedure (including time to storage) | **Y** | Included in main manuscript under "Sample Collection and Processing" |
| Sample aliquotation, storage conditions and duration | **Y** | Included in main manuscript under "Sample Collection and Processing" |
| **2. NUCLEIC ACID EXTRACTION** | | |
| Description of extraction method including amount of sample processed | **Y** | Included in main manuscript under "Total Nucleic Acid Extraction and Reverse Transcription" |
| Volume of solvent used to elute/resuspend extract | **Y** | Included in main manuscript under "Total Nucleic Acid Extraction and Reverse Transcription" |
| Number of extraction replicates | **Y** | Included in main manuscript under "Total Nucleic Acid Extraction and Reverse Transcription" |
| Extraction blanks included? | **Y** | Included in main manuscript under "Quality Control Elements" |
| **3. NUCLEIC ACID ASSESSMENT AND STORAGE** | | |
| Method to evaluate quality of nucleic acids | **N** | Previous assessments deem this nucleic acid to be of suitable quality for the purpose |
| Method to evaluate quantity of nucleic acids (including molecular weight and calculations when using mass) | **N** | Previous assessments deem this nucleic acid to be of suitable quality for the purpose |
| Storage conditions: temperature, concentration, duration, buffer, aliquots | **Y** | Included in main manuscript under "Total Nucleic Acid Extraction and Reverse Transcription" |
| Clear description of dilution steps used to prepare working DNA solution | **N** | No further dilutions steps were performed for cDNA |
| **4. NUCLEIC ACID MODIFICATION** | | |
| Template modification (digestion, sonication, pre-amplification, bisulphite etc.) | **NA** | NA |
| Details of repurification following modification if performed | **NA** | NA |
| **5. REVERSE TRANSCRIPTION** | | |
| cDNA priming method and concentration | **Y** | Included in main manuscript under "Total Nucleic Acid Extraction and Reverse Transcription" |
| One or two step protocol (include reaction details for two step) | **Y** | Included in main manuscript under "Total Nucleic Acid Extraction and Reverse Transcription" |
| Amount of RNA added per reaction | **Y** | Included in main manuscript under "Total Nucleic Acid Extraction and Reverse Transcription" |
| Detailed reaction components and conditions | **Y** | Included in main manuscript under "Total Nucleic Acid Extraction and Reverse Transcription" |
| Estimated copies measured with and without addition of RT* | **N** | No RT control was eliminated after several hundred assays with this method showed no amplification |
| Manufacturer of reagents used with catalogue and lot numbers | **Y** | Included in main manuscript under "Total Nucleic Acid Extraction and Reverse Transcription"; catalog and lot numbers not provided |
| Storage of cDNA: temperature, concentration, duration, buffer and aliquots | **Y** | Included in main manuscript under "Total Nucleic Acid Extraction and Reverse Transcription" |
| **6. dPCR OLIGONUCLEOTIDES DESIGN AND TARGET INFORMATION** | | |
| Sequence accession number or official gene symbol | **Y** | Table S3 |
| Method (software) used for design and *in silico* verification | **Y** | References are included in Table S3 |
| Location of amplicon | **Y** | Table S3 |
| Amplicon length | **Y** | Table S3 |
| Primer and probe sequences (or amplicon context sequence)** | **Y** | Table S3 |
| Location and identity of any modifications | **Y** | Table S3 |
| Manufacturer of oligonucleotides | **Y** | Table S3 |
| **7. dPCR PROTOCOL** | | |
| Manufacturer of dPCR instrument and instrument model | **Y** | Included in main manuscript under "Digital PCR Fluorescence Amplitude Thresholding" |
| Buffer/kit manufacturer with catalogue and lot number | **Y** | Included in main manuscript under "Data Analysis and Normalization"; catalog and lot number not included |
| Primer and probe concentration | **Y** | Included in main manuscript under "Data Analysis and Normalization" |
| Pre-reaction volume and composition (incl. amount of template and if restriction enzyme added) | **Y** | Included in main manuscript under "Data Analysis and Normalization" |
| Template treatment (initial heating or chemical denaturation) | **Y** | Included in main manuscript under "Data Analysis and Normalization" |
| Polymerase identity and concentration, Mg++ and dNTP concentrations*** | **N** | Proprietary composition |
| Complete thermocycling parameters | **Y** | Tables S6, S7 |
| **8. ASSAY VALIDATION** | | |
| Details of optimization performed | **N** | Assays previously optimized in Beattie, et al., 2022 |
| Analytical specificity (vs. related sequences) and limit of blank (LOB) | **Y** | Included in main manuscript under "Limit of Blank and Process Limit of Detection" |
| Analytical sensitivity/LoD and how this was evaluated | **Y** | Included in main manuscript under "Limit of Blank and Process Limit of Detection" |
| Testing for inhibitors (from biological matrix/extraction) | **Y** | Included in main manuscript under "Quality Control Elements" |
| **9. DATA ANALYSIS** | | |
| Description of dPCR experimental design | **Y** | Figure S2, Tables S3, S5 |
| Comprehensive details negative and positive of controls (whether applied for QC or for estimation of error) | **Y** | Included in main manuscript under "Quality Control Elements" |
| Partition classification method (thresholding) | **Y** | Included in main manuscript under "Digital PCR Fluorescence Amplitude Thresholding" |
| Examples of positive and negative experimental results (including fluorescence plots in supplemental material) | **Y** | Included in main manuscript under "BioRad Droplet Digital PCR" and "QIAGEN QIAcuity One Digital PCR " |
| Description of technical replication | **Y** | Included in main manuscript under "BioRad Droplet Digital PCR" and "QIAGEN QIAcuity One Digital PCR " |
| Repeatability (intra-experiment variation) | **Y** | Included in main manuscript under "Results" |
| Reproducibility (inter-experiment/user/lab etc. variation) | **Y** | Included in main manuscript under "Results" |
| Number of partitions measured (average and standard deviation) | **Y** | Included in main manuscript under "Results" |
| Partition volume | **Y** | Included in main manuscript under "Bio-Rad QX200 Droplet Digital PCR" and "QIAGEN QIAcuity Four Digital PCR " |
| Copies per partition (λ or equivalent) (average and standard deviation) | **N** | Not included in analysis |
| dPCR analysis program (source, version) | **Y** | Included in main manuscript under "Data Analysis and Normalization" |
| Description of normalization method | **Y** | Included in main manuscript under "Data Analysis and Normalization" |
| Statistical methods used for analysis | **Y** | Included in main manuscript under "Statistical Analysis" |
| Data transparency | **Y** | Included in supplementary materials |
